# Clinical Characteristics of Subependymal Giant Cell Astrocytoma in Tuberous Sclerosis Complex

**DOI:** 10.3389/fneur.2019.00705

**Published:** 2019-07-03

**Authors:** Anna C. Jansen, Elena Belousova, Mirjana P. Benedik, Tom Carter, Vincent Cottin, Paolo Curatolo, Maria Dahlin, Lisa D'Amato, Guillaume Beaure d'Augères, Petrus J. de Vries, José C. Ferreira, Martha Feucht, Carla Fladrowski, Christoph Hertzberg, Sergiusz Jozwiak, John A. Lawson, Alfons Macaya, Ruben Marques, Rima Nabbout, Finbar O'Callaghan, Jiong Qin, Valentin Sander, Matthias Sauter, Seema Shah, Yukitoshi Takahashi, Renaud Touraine, Sotiris Youroukos, Bernard Zonnenberg, John C. Kingswood

**Affiliations:** ^1^Pediatric Neurology Unit, Department of Pediatrics, Universitair Ziekenhuis Brussel, Vrije Universiteit Brussel, Brussels, Belgium; ^2^Research and Clinical Institute of Pediatrics, Pirogov Russian National Research Medical University, Moscow, Russia; ^3^Child Neurology Department, SPS Pediatrična Klinika, Ljubljana, Slovenia; ^4^Tuberous Sclerosis Association, Nottingham, United Kingdom; ^5^Hôpital Louis Pradel, Claude Bernard University Lyon 1, Lyon, France; ^6^Child Neurology and Psychiatry Unit, Systems Medicine Department, Tor Vergata University Hospital, Rome, Italy; ^7^Neuropediatric Department, Astrid Lindgren Childrens Hospital, Stockholm, Sweden; ^8^Novartis Farma S.p.A., Origgio, Italy; ^9^Association Sclérose Tubéreuse de Bourneville, Gradignan, France; ^10^Division of Child and Adolescent Psychiatry, University of Cape Town, Cape Town, South Africa; ^11^Neurology Department, Centro Hospitalar Lisboa Ocidental, Lisbon, Portugal; ^12^Medical University of Vienna, Universitätsklinik für Kinder-und Jugendheilkunde, Vienna, Austria; ^13^Associazione Sclerosi Tuberosa ONLUS, Milan, Italy; ^14^European Tuberous Sclerosis Complex Association, In den Birken, Dattein, Germany; ^15^Zentrum für Neuropädiatrie und Sozialpädiatrie, Vivantes-Klinikum Neukölln, Berlin, Germany; ^16^Department of Child Neurology, Warsaw Medical University, Warsaw, Poland; ^17^Department of Neurology and Epileptology, The Children's Memorial Health Institute, Warsaw, Poland; ^18^The Tuberous Sclerosis Multidisciplinary Management Clinic, Sydney Children's Hospital, Randwick, NSW, Australia; ^19^Pediatric Neurology Section, Hospital Universitari Vall d'Hebron, Barcelona, Spain; ^20^Institute of Biomedicine, University of Leon, León, Spain; ^21^Department of Pediatric Neurology, Necker Enfants Malades Hospital, Paris Descartes University, Paris, France; ^22^Institute of Child Health, University College London, London, United Kingdom; ^23^Department of Pediatrics, Peking University People's Hospital, Beijing, China; ^24^Neurology and Rehabilitation, Tallinn Children Hospital, Tallinn, Estonia; ^25^Klinikum Kempten, Klinikverbund Kempten-Oberallgäu gGmbH, Kempten, Germany; ^26^Novartis Healthcare Pvt. Ltd., Hyderabad, India; ^27^National Epilepsy Center, Shizuoka Institute of Epilepsy and Neurological Disorders, NHO, Shizuoka, Japan; ^28^Department of Genetics, CHU-Hôpital Nord, Saint Etienne, France; ^29^First Department of Paediatrics, St. Sophia Children's Hospital, Athens University, Athens, Greece; ^30^Department of Internal Medicine, University Medical Center, Utrecht, Netherlands; ^31^Cardiology Clinical Academic Group, Molecular and Clinical Sciences Research Centre, St. Georges University of London, London, United Kingdom

**Keywords:** mTOR, registry, SEGA, TOSCA, tuberous sclerosis complex

## Abstract

**Background:** This study evaluated the characteristics of subependymal giant cell astrocytoma (SEGA) in patients with tuberous sclerosis complex (TSC) entered into the TuberOus SClerosis registry to increase disease Awareness (TOSCA).

**Methods:** The study was conducted at 170 sites across 31 countries. Data from patients of any age with a documented clinical visit for TSC in the 12 months preceding enrollment or those newly diagnosed with TSC were entered.

**Results:** SEGA were reported in 554 of 2,216 patients (25%). Median age at diagnosis of SEGA was 8 years (range, <1–51), with 18.1% diagnosed after age 18 years. SEGA growth occurred in 22.7% of patients aged ≤ 18 years and in 11.6% of patients aged > 18 years. SEGA were symptomatic in 42.1% of patients. Symptoms included increased seizure frequency (15.8%), behavioural disturbance (11.9%), and regression/loss of cognitive skills (9.9%), in addition to those typically associated with increased intracranial pressure. SEGA were significantly more frequent in patients with *TSC2* compared to *TSC1* variants (33.7 vs. 13.2 %, *p* < 0.0001). Main treatment modalities included surgery (59.6%) and mammalian target of rapamycin (mTOR) inhibitors (49%).

**Conclusions:** Although SEGA diagnosis and growth typically occurs during childhood, SEGA can occur and grow in both infants and adults.

## Introduction

Tuberous sclerosis complex (TSC) is an autosomal dominant genetic disorder characterized by growth of hamartomas in several organs, including the brain, kidneys, lungs, heart, eyes, and skin (1). Subependymal giant cell astrocytomas (SEGA) are benign, non-infiltrative brain lesions classified by the World Health Organization as grade I, characteristically observed in patients with TSC (2, 3). They are typically slow-growing tumours composed of different cell lineages and are not purely astrocytic in nature (4). Historically, SEGA diagnosis was based on histology (5), but over time, diagnosis became imaging based. In 2013, an international panel of experts defined the imaging characteristics of SEGA as a lesion at the caudothalamic groove with either a size of >1 cm in any direction or a subependymal lesion at any location that has shown serial growth on consecutive imaging regardless of size. Most SEGA show clear enhancement after contrast administration. However, a growing subependymal lesion even in the absence of enhancement should be considered a SEGA (6). The prevalence of SEGA was previously reported to range from 4 to 20% (2, 7–11). The studies mentioned were based on relatively small patient numbers. In the largest series by Adriaensen et al. evaluating 214 patients with TSC, SEGA was defined as a subependymal lesion near the foramen of Monro showing contrast enhancement after administration of intravenous gadolinium. SEGA occurred in 20% of individuals in this study and average maximum SEGA size was 11.4 mm (range, 4–29 mm) (2).

Although SEGA are histologically benign, their location near the foramen of Monro and their tendency to grow can lead to obstructive hydrocephalus with consecutive substantial morbidity and mortality (12). Symptoms associated with growing SEGA include those typically associated with raised intracranial pressure (headaches, photophobia, diplopia, ataxia, seizures) and/or detrimental effects on cognition and/or increased seizure burden, learning, or behaviour (13). SEGA typically appear in the first 2 decades of life, with a mean age at presentation below 18 years (14). However, there have been reports of SEGA detection prenatally (as early as at 19 weeks gestation) (15–17), as well as new diagnoses after 20 years of age (2, 18). There have been prior reports suggesting that SEGA occur at a younger age in patients with *TSC2* mutations compared with those with *TSC1* mutations (8, 19).

Currently, surgical resection and mammalian target of rapamycin (mTOR) inhibitors are the recommended treatment options for SEGA associated with TSC. Surgical resection should be considered for acutely symptomatic SEGA, while either surgical resection or medical treatment with mTOR inhibitors may be considered for growing, but not acutely symptomatic SEGA (20). However, surgical resection may be associated with preoperative and postoperative complications, and incompletely resected SEGA often tend to regrow (6, 14, 21). Everolimus, an inhibitor of mTOR, the central pathway involved in the pathophysiology of TSC, has been approved by the Food and Drug Administration (FDA) and European Medicines Agency (EMA) for patients with TSC-associated SEGA who require therapeutic intervention, but are not candidates for surgical resection (14). mTOR inhibitors have also shown improvements in the other manifestations of TSC including renal angiomyolipomas, epilepsy, lymphangioleiomyomatosis, and facial angiofibromas (22–25).

Although substantial progress has been made in our understanding of the biological and genetic basis of TSC in the past decade, several questions, especially those related to the natural history of the disease, remain unanswered. To address this gap, the TOSCA (**T**uber**O**us **SC**lerosis registry to increase disease **A**wareness) registry was designed with the aim of providing deeper insights into the manifestations of TSC and its management. The baseline core data of the TOSCA registry published previously provided understanding of the overall manifestations and natural history of TSC (26). Here, we present the clinical characteristics of SEGA in children and adults.

## Patients and Methods

TOSCA is a non-interventional, multicenter, international natural history study conducted at 170 sites across 31 countries. The study design and methodology of TOSCA have been described in detail previously (27). In brief, between August 2012 and August 2014, patients of any age with a documented clinic visit for TSC in the 12 months preceding enrollment or those newly diagnosed with TSC were enrolled. General information on patient background, such as demographic data, family history, genotype, vital signs, prenatal history, clinical features of TSC across all organ systems, comorbidities, and rare manifestations, was collected at baseline and at regular visits scheduled at a maximum interval of 1 year. Follow-up visits were scheduled according to the standard practice of the site and as per the treating physician's best judgement. The data were recorded on an electronic case report form (eCRF) that was accessed via a secure web portal hosted by a contract research organization. Input of data was carried out by local investigators or their deputies, and then independently checked by a network of clinical research associates for accuracy and consistency using the original local case records. The web portal has an explanatory manual to guide the investigators.

Data collected specific to SEGA included tumour characteristics such as presence of single or multiple SEGA, clinical signs and symptoms associated with SEGA, and management. Characteristics of SEGA according to the age at consent were evaluated. The study also assessed the association between genotype (*TSC1* vs. *TSC2*) and SEGA characteristics using Chi-square test or fisher exact test, and median test. Since baseline data were collected prior to the 2013 international consensus on SEGA definition, no specific inclusion criteria were defined. The TOSCA cohort therefore reflects worldwide clinical practice.

Given that the natural history study is exploratory in nature, background and clinical parameters were reported with descriptive statistics only. All eligible patients enrolled in the TOSCA registry were considered for analysis. Categorical data were reported as frequencies and percentages, and continuous variables were expressed as mean (± standard deviation) or as median (range), unless stated otherwise.

TOSCA was designed and conducted according to the Guidelines for Good Clinical Practice and ethical principles outlined in the Declaration of Helsinki (28, 29). After appropriate approval by central and all local human research ethics committees, written informed consent was obtained from all patients, parents, or guardians prior to enrollment.

## Results

As of September 30, 2015, 2,216 patients (1,154 females and 1,062 males) with TSC were enrolled in the TOSCA registry from 170 sites across 31 countries. The demographic and clinical characteristics of the enrolled patients are shown in Table 1. The majority of these patients (70%) were enrolled by pediatric or adult neurologists.

**Table 1:** Demographics and clinical characteristics of participants in the TOSCA study (*N* = 2,216).

**Characteristics**	**Baseline data**
Age at diagnosis of TSC, years; median (range)	1 (<1–69)
Gender, *n* (%)	
Male	1,062 (47.9)
Female	1,154 (52.1)
Patients with molecular testing, *n* (%)	1,000 (45.1)
Genetic testing, *n* (%)^a^	
No mutation identified	144 (14.4)
*TSC1* mutation^b^	197 (19.7)
*TSC2* mutation^b^	644 (64.4)
Both *TSC1* and *TSC2* mutations	6 (0.6)
Variation type, *n* (%)^c^	
Pathogenic mutation	678 (67.8)
Variant of unknown significance	66 (6.6)
Patients with prenatal diagnosis, *n* (%)	144 (6.5)

*TSC, tuberous sclerosis complex; TOSCA, TuberOus SClerosis registry to increase disease Awareness*.

a *Information on the type of mutation was missing for 9 patients*.

b *The count (n) includes 6 patients who had both TSC1 and TSC2 mutations*.

c *The count (n) includes 23 patients who had both variation types*.

**Figure 1:**
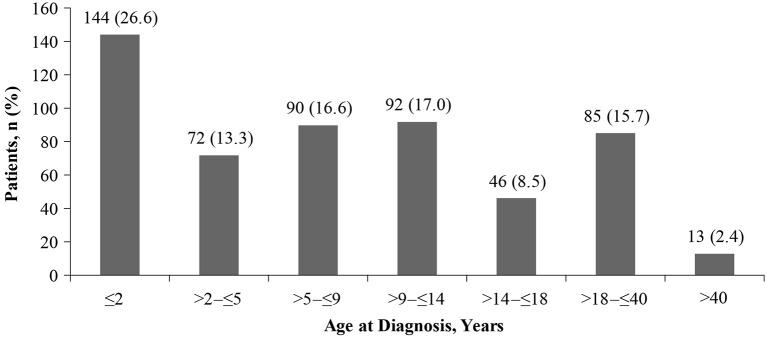
Proportion of patients with SEGAs according to age at SEGA diagnosis (*n* = 542).

Overall, SEGA were reported in 554 patients (25%); 275 (49.6%) were males and 279 (50.4%) were females. Of these, SEGA were present at baseline in 463 patients (83.6%), resolved with treatment before baseline in 80 patients (14.4%), and were reported to have resolved spontaneously in 10 patients (1.8%), the latter possibly due to measurement errors in small lesions. Detailed information was lacking for one patient. The median age at SEGA diagnosis was 8 years (range, <1–51 years). SEGA were diagnosed before 2 years of age in 26.6%, before 18 years in 81.9% of patients, and after 18 years in 18.1% patients (Figure 1). The oldest patient diagnosed with SEGA in the TOSCA cohort was 51 years.

Of the 463 patients with SEGA at baseline, 209 (45.1%) had multiple SEGA and in 208 patients (44.9%) SEGA were present bilaterally (Table 2). Among patients with SEGA present at the at the time of baseline visit, SEGA growth was observed in 68 out of 300 patients aged ≤ 18 years (22.7%) and 19 out of 163 patients aged > 18 years (11.6%). In total, 87 out of 463 patients showed SEGA growth since previous scan (18.8%). Of these, 7 patients (8%) were aged <2 years, 68 patients (78.2%) were aged ≤ 18 years, while 19 patients (21.8%) were aged > 18 years. The median time between consecutive scans was 1 year (mean 1.5 years, range <1–18). At the time of assessment, 321 patients (69.3%) were asymptomatic. Of these, 29 (9.0%) were aged <2 years, 175 (54.5%) were > 2 years and ≤ 18 years, and 117 (36.4%) were aged > 18 years (Table 3). One or more symptoms (alone or in combination) assigned to SEGA in our cohort were observed in 233 patients (50.3%). The most frequent symptoms were increased seizure frequency in 73 patients (15.8%), behavioural disturbance in 55 (11.9%), regression/loss of cognitive skills in 46 (9.9%), and headache in 39 (8.4%) (Table 2).

**Table 2:** Clinical characteristics of SEGA at baseline visit in overall population and according to mutation type.

	**Overall** **(*N* = 2,216)**	**Patients with *TSC2* mutation (*n* = 644)**	**Patients with *TSC1* mutation (*n* = 197)**	***p*-value^c^**
Patients with a history of SEGA^a^	554 (25.0)	217 (33.7)	26 (13.2)	<0.0001
Median age at diagnosis, years^b^; median (range)	8 (<1–51)	7.0 (<1–49)	7.0 (<1–51)	0.6167
No. of patients diagnosed with SEGA at < age 2 years^a^	144 (26.6)	67 (31.2)	5 (20.8)	0.3812
No. of patients with SEGA present at the time of visit, *n*^a^	463	185	20	0.2472
Multiple	209 (45.1)	90 (48.6)	8 (40.0)	0.8368
Bilateral	208 (44.9)	84 (45.4)	7 (35.0)	0.9550
Growing SEGA since previous scan	87 (18.8)	35 (18.9)	1 (5.0)	0.3302
Signs and symptoms assigned to SEGA^a^				
None	321 (69.3)	125 (67.6)	11 (55.0)	0.1960
Increase in seizure frequency	73 (15.8)	38 (20.5)	4 (20.0)	1.0000
Behavioural disturbance	55 (11.9)	25 (13.5)	3 (15.0)	0.7311
Regression/loss of cognitive skills	46 (9.9)	20 (10.8)	1 (5.0)	0.6996
Headache	39 (8.4)	15 (8.1)	4 (20.0)	0.0854
Ventriculomegaly	25 (5.4)	9 (4.9)	1 (5.0)	1.0000
Increased intracranial pressure	24 (4.6)	8 (4.3)	3 (15.0)	0.0710
Sleep disorder	14 (3.0)	7 (3.8)	0	1.0000
Eye movement abnormalities	13 (2.8)	6 (3.2)	1 (5.0)	0.5028
Visual impairment	8 (1.7)	4 (2.2)	0	1.0000
Papilledema	8 (1.7)	5 (2.7)	1 (5.0)	0.4498
Neuroendocrine dysfunction	6 (1.3)	3 (1.6)	0	1.0000
Other	14 (3.0)	5 (2.7)	2 (10.0)	0.1313

a *Chi-square or Fisher exact test*.

b *Median test showing comparison of SEGA characteristics between those with TSC1 mutations and TSC2 mutations*.

c *TSC1 vs. TSC2 at baseline*.

*SEGA, subependymal giant cell astrocytoma*.

**Table 3:** Clinical characteristics of SEGA at baseline visit according to age categories.

	**Age at TOSCA consent, years**						
	**≤2** **(*n* = 283)**	**>2–≤5** **(*n* = 301)**	**>5–≤9** **(*n* = 335)**	**>9–≤14** **(*n* = 307)**	**>14–≤18** **(*n* = 184)**	**>18–≤40** **(*n* = 579)**	**>40** **(*n* = 227)**
Patients with a history of SEGA	43 (15.2)	51 (16.9)	98 (29.3)	98 (31.9)	68 (37.0)	167 (28.8)	29 (12.8)
No. of patients with SEGA present at the time of visit, *n*	41 (14.5)	45 (15.0)	82 (24.5)	78 (25.4)	54 (29.3)	139 (24.0)	24 (10.6)
Multiple	14 (4.9)	13 (4.3)	35 (10.4)	31 (10.1)	20 (10.9)	53 (9.2)	6 (2.6)
Bilateral	13 (4.6)	13 (4.3)	33 (9.9)	31 (10.1)	20 (10.9)	51 (8.8)	9 (4.0)
Growing SEGA since previous scan	7 (2.5)	9 (3.0)	19 (5.7)	19 (6.2)	14 (7.6)	19 (3.3)	0
Signs and symptoms							
None	29 (10.2)	37 (12.3)	61 (18.2)	48 (15.6)	29 (15.8)	97 (16.8)	20 (8.8)
Increase in seizure frequency	8 (2.8)	7 (2.3)	10 (3.0)	13 (4.2)	12 (6.5)	22 (3.8)	1 (0.4)
Behavioural disturbance	3 (1.1)	3 (1.0)	13 (3.9)	10 (3.3)	5 (2.7)	20 (3.5)	1 (0.4)
Regression/loss of cognitive skills	5 (1.8)	3 (1.0)	6 (1.8)	8 (2.6)	9 (4.9)	14 (2.4)	1 (0.4)
Headache	0	1 (0.3)	3 (0.9)	8 (2.6)	10 (5.4)	15 (2.6)	2 (0.9)
Ventriculomegaly	3 (1.1)	0	4 (1.2)	7 (2.3)	4 (2.2)	7 (1.2)	0
Increased intracranial pressure	0	1 (0.3)	2 (0.6)	5 (1.6)	6 (3.3)	8 (1.4)	2 (0.9)
Sleep disorder	2 (0.7)	2 (0.7)	0	6 (2.0)	0	4 (0.7)	0
Eye movement abnormalities	1 (0.4)	1 (0.3)	1 (0.3)	3 (1.0)	2 (1.1)	5 (0.9)	0
Visual impairment	0	0	2 (0.6)	1 (0.3)	1 (0.5)	4 (0.7)	0
Papilledema	0	0	1 (0.3)	1 (0.3)	2 (1.1)	3 (0.5)	1 (0.4)
Neuroendocrine dysfunction	0	0	2 (0.6)	0	1 (0.5)	3 (0.5)	0
Other	0	0	2 (0.6)	6 (2.0)	1 (0.5)	5 (0.9)	0

*Percentages were calculated using number of patients in each age group as denominator. SEGA, subependymal giant cell astrocytoma*.

The characteristics of SEGA associated with mutations in *TSC1* and *TSC2* are shown in Table 2. SEGA were significantly more frequently observed in patients with a *TSC2* mutation compared to those with a *TSC1* mutation (33.7 vs. 13.2%, *p* < 0.0001). However, there was no significant difference with respect to SEGA diagnosis before 2 years of age (*p* = 0.3812), multiple (*p* = 0.8368), bilateral (*p* = 0.9550) or growing SEGA (*p* = 0.3302), and presence of SEGA-related symptoms (*p* > 0.05) in patients with mutations in *TSC1* compared to *TSC2* (Table 2). A total of 208 patients received at least one treatment after SEGA diagnosis with a median time from SEGA diagnosis to treatment of 319 days (range, 1–5517 days). The most common treatment modalities included surgical resection (124 patients, 59.6%), mTOR inhibitors (102 patients, 49%), and ventriculoperitoneal shunt (22 patients, 10.6%), used alone or in combination.

## Discussion

Together with cortical tubers, white matter radial migration lines, and subependymal nodules, SEGA represent one of the three major central nervous system features in the diagnostic criteria for TSC (30). Although benign and slow growing, SEGA are potentially lethal and can cause serious neurological complications including raised intracranial pressure due to obstructive hydrocephalus (7). However, to date, studies on the natural history of SEGA and TSC have been sparse, smaller in scale, and typically from a single centre (6). The TOSCA disease registry has collected disease information on the largest cohort of patients with TSC to date.

In the current study, SEGA was reported in 25% of patients with TSC enrolled in the study; of whom, ~45% had bilateral SEGA. Most studies have reported lower rates of SEGA in patients with TSC ranging from 4 to 20% (2, 7–11). The method used for diagnosis of SEGA in these studies varied substantially. The highest rates reported to date came from a case series of 214 patients with TSC, which reported SEGA in 20% of their patients (2). In this study, SEGA was defined as a subependymal lesion near the foramen of Monro showing contrast enhancement after administration of intravenous gadolinium. No specifications on size or growth were taken into consideration, which is in line with the TOSCA cohort. Most of the patients in TOSCA were enrolled from specialist neurology centres, which might have influenced the number of patients with SEGA included in TOSCA. We also have no data on the number of patients who declined to participate in TOSCA. It cannot be excluded that patients with milder disease were less likely to participate. In addition, patient with milder disease might be less likely to have SEGA, potentially contributing to selection bias.

Published data reported a preponderance of SEGA in children and adolescents (2, 4, 7, 10). In TOSCA, most SEGA were indeed diagnosed in childhood, with a median age at SEGA diagnosis of 8 years. Importantly, 26.6% of patients were diagnosed with SEGA before 2 years of age (Figure 1), and growing SEGA were observed in 2.5% of patients aged <2 years (Table 3), highlighting the need for early monitoring. The potential occurrence of early SEGA growth has been highlighted previously. The study reported SEGA surgery before the age of 3 years in 9.4% of total 57 children enrolled in the study (31).

Prior reports of SEGA growth after the age of 25 years have been very rare (32). Surprisingly, we identified growing SEGA in 19 patients (2.4%) beyond the age of 18 years. This underlines the need to remain vigilant in adult patients with known SEGA as pointed out in the international recommendations for the surveillance and management of TSC (6, 20). The international consensus panel recommended performing brain imaging every 1–3 years until the age of 25 years. In TOSCA, the median time between scans for SEGA follow-up was 1 year (range, 0–18 years), which is in line with the international recommendations (6, 20). The frequency of scans within the recommended range of every 1–3 years needs to be determined based on clinical grounds, with scans performed more frequently in asymptomatic SEGA patients who are younger, whose SEGA are larger or growing, or who have developmental delays or intellectual disability. Individuals without SEGA by the age of 25 years seem not to need continued imaging (20). For those with SEGA at age 25 years, follow-up MRI intervals may be increased provided the patient remains clinically stable.

New onset of symptoms related to raised intracranial pressure as well as increase in seizure frequency or change in neurological status and behaviour or loss of skills (especially in patients with intellectual disability) should trigger an earlier scan. Similarly, a growing SEGA should prompt a more frequent clinical and radiological follow-up. Parents and patients should be educated regarding relevant symptoms that should prompt referral to medical evaluation (6). The TOSCA data suggest that SEGA-related symptoms (especially early symptoms) are not exclusively limited to signs of increased intracranial pressure.

Previous studies suggested that *TSC2* mutations are associated with a more severe clinical phenotype (8, 19). Findings from TOSCA confirmed that SEGA were present more frequently in patients with mutations in *TSC2* compared to *TSC1*.However, differences in age at onset, SEGA growth or SEGA-related symptoms were not significant. The reason for this observation remains unclear.

In the current study, surgical resection (59.6%) and mTOR inhibitor (49%) were the most common treatment modalities at baseline. Current international recommendations propose the use of surgical resection for acutely symptomatic SEGAs. For growing but asymptomatic SEGA, both surgical resection and mTOR inhibitors are potential treatments. In determining the best option, discussion of the complication risks, adverse effects, cost, length of treatment, family preference, surgical expertise in SEGA, and potential impact on TSC-associated comorbidities should be included in the decision-making process (20, 33). mTOR inhibitors have been shown to be effective in the treatment of other TSC manifestations including epilepsy, renal angiomyolipoma, and lymphangioleiomyomatosis (22–25). Hence, the treatment with mTOR inhibitors may be preferred over surgery in patients with multiple organ involvement or with a combination of mTOR inhibitor-responsive lesions. mTOR inhibitors are also recommended for patients with large or bilateral SEGA that are not amenable to surgical resection (33). SEGA are likely to regrow in case of incomplete resection. This was illustrated in a study of 57 patients with TSC who underwent a total of 64 SEGA surgeries. Gross total resection was performed in 58 cases with no regrowth, while 5 out of 6 children who underwent partial resection showed tumour regrowth within 3–12 months (31). It is also important to consider that long-term mTOR inhibitor treatment may be required, as discontinuation of mTOR inhibitors is typically associated with regrowth of tumours (21).

The median time from SEGA diagnosis to treatment initiation was 319 days. This likely reflects a watch and wait approach to document growth and the need for intervention.

The current study has the following limitations: firstly, the observational nature allowed collection of only those data that were already available from clinical practice and hence reflects “real world” data. Secondly, a major challenge for this registry was to ensure that data about all the disease manifestations for each patient were reported although the sites involved in the registry did not always follow patients for all disease manifestations in the same way. However, the low number of missing data for SEGA (4.7%) reflects good quality of data collection.

## Conclusion

In summary, the study highlights that the rates of SEGA in patients with TSC might be higher than previously reported. Increase in seizure frequency, behavioural disturbance, regression/loss of cognitive skills were identified as frequent symptoms associated with SEGA, over and above headaches, typically associated with raised intracranial pressure. SEGA may already be present and grow at a very young age. Although SEGA mostly occur in childhood, it is important to be vigilant in adults as well, since SEGA growth does occur also in these age groups.

## Data Availability

Novartis supports the publication of scientifically rigorous analysis that is relevant to patient care, regardless of a positive or negative outcome. Qualified external researchers can request access to anonymized patient-level data, respecting patient informed consent, contacting study sponsor authors. The protocol can be accessed through EnCePP portal http://www.encepp.eu/ (EU PAS Register Number EUPAS3247).

## Ethics Statement

The study protocol and all amendments were reviewed and approved (if applicable) by independent ethics committee/institutional review board for each centre: National Hospital Organization Central Ethics Committee; Gazi University Clinical Research Ethics Committee; Independent Multidisciplinary Committee on Ethical Review of Clinical Trials; Peking Union Medical College Hospital; Commissie Medische Ethiek UZ Brussel; CNIL (Commission National de l'Informatique et des Libertés), CCTIRS (Comité Consultatif sur le traitement de l'information en matière de recherche dans le domaine de la santé); Comité Etico Investigación Clínica de Euskadi (CEIC-E); Consejeria de Salud y Bienestar Social, Dirección General de Calidad, Investigación, Desarrollo e Innovación, Comité Coordinador de Ética de la Investigación Biomédica de Andalucía; Research Ethics Committee of the University of Tartu (UT REC); Ethikkommission der Medizinischen Universität Graz; North Wales REC—West; Regionala Etikprövningsnämnden i Göteborg; REK—Regionale komiteer for medisinsk og helsefaglig forskningsetikk; Komisja Bioetyczna przy Instytucie Pomnik Centrum Zdrowia Dziecka; Ethikkommission bei der Ludwig-Maximilians-Universitat München; Hokkaido University Hospital Independent clinical research Institutional Ethics Committee; Medical Juntendo University Institutional Ethics Committee; National Center for Chile Health and Deveropment of IRB; Osaka University Hospital of IRB; Ethics Committee at Moscow Institute of Pediatrics and Pediatric Surgery; Peking University First Hospital; Sanbo Brain Hospital Capital Medical University; Tianjin Children's Hospital; Childrens Hospital Of Fudan University; Zhongshan Hospital Fudan University; Fudan University Shanghai Cancer Center; The Second Affiliated Hospital of Guangzhou Medical University; The First Affiliated Hospital, Sun Yan-Sen University; The First Affiliated Hospital Of Guangzhou Medical University; Shenzhen Children's Hospital; West China Hospital, Sichuan University; Xijing Hospital; Children's Hospital of Chongqing Medical University; Wuhan Children's Hospital; The second affiliated hospital of Xi'an jiaotong university; Guangdong 999 brain hospital; Seoul National University Hospital Institutional Review Board; National Taiwan University Hospital (NTUH) Research Ethics Committee (REC); Institutional Review Board of the Taichung Veterans General Hospital; Institutional Review Board of Chung Shan Medical University Hospital; Institutional Review Board, Tungs' Taichung MetroHarbor Hospital; Institutional Review Board of National Cheng Kung University Hospital; Metro South Human Research Ethics Committee; Sydney Children's Hospital Network Human Research Ethics Committee; St. Vincents Hospital Human Research Ethics Committee; Royal Melbourne Hospital Human Research Ethics Committee; Siriraj Institutional Review Board; The Institutional Review board, Faculty of Medicine, Chulalongkorn University, Third Floor, Ananthamahidol Building, King Chulalongkorn Memorial Hospital; The committee on Human Rights Related to Research Involving Human Subjects; Institutional Review board, Royal Thai Army Medical Department IRB RTA, Fifth Floor, Phramongkutklaowejvitya Building, Phramongkutklao College of Medicine; Research Ethics Committee, Faculty of Medicine, Chiang Mai University; Research and Development, Queen Sirikit National Institute of Child Health; Human Research Ethics Committee, Faculty of Health Sciences, University of Cape Town; Shaare Zedek Meidcla center Helsinki comittee; Sheba Medical center Helsinki comittee; Tel Aviv Sourasly Medical center Helsinki comittee; General University Hospital of Patras Ethics Committee; Pendeli Children's Hospital Ethics Committee; General University Hospital of Athens 'G. Gennimatas Ethics Committee; Evaggelismos General Hospital Ethics Committee; General University Hospital of Thessaloniki AHEPA Ethics Committee; General University Hospital of Ionnina Ethics Committee; METC UMC Utrecht; Direcció General de Regulació, Planificació i Recursos Sanitaris; Comité Ético de Investigación Clínica del Hospital Universitario Vall d'Hebron de Barcelona, Generalitat de Catalunya.Departament de Salut; Comité Ético de Investigación Clínica Hospital Universitario La Paz; Dirección General de Ordenación e Inspección, Consejería de Sanidad Comunidad de Madrid, Servicios de Control Farmacéutico y Productos Sanitarios; Comité Etico Investigación Clínica del Hospital Universitario y Politécnico de La Fe; Dirección General de Farmàcia i Productes Sanitaris, Generalitat de Valencia; Comité de Ética de la Investigación de Centro de Granada; Instituto Aragonés de Ciencias de la Salud (IACS); Comité Etico Investigación Clínica Regional del Principado de Asturias; Comité Etico Investigación Clínica Hospital 12 de Octubre; Comité Etico Investigación Clínica Hospital Universitario Virgen de la Arrixaca; Sección de Ordenación e Inspección Farmacéutica Departamento de Salud; Comité Ético de Investigación Clínica del Hospital Universitario del Río Hortega de Valladolid; Comissão de Ética para a Saúde (CES), Centro Hospitalar de Lisboa Ocidental, EPE; Comissão de Ética para a Saúde (CES), Centro Hospitalar do Porto, E.P.E; Comissão de Ética para a Saúde (CES), Centro Hospitalar Lisboa Central, EPE; Comissão de Ética para a Saúde (CES), Hospital Garcia de Orta, EPE; Comissão de Ética para a Saúde (CES), Centro Hospitalar de São João, EPE; Comissão de Ética para a Saúde (CES), Hospital Professor Doutor Fernando Fonseca, EPE; Comissão de Ética para a Saúde (CES), Centro Hospitalar do Algarve, EPE (Unidade de Faro); LUHS Kaunas Regional Biomedical Research Ethics Committee; Paula Stradiņa klīniskās universitātes slimnīcas, Attīstības biedrības Klīniskās izpētes Ētikas komiteja, Ethics Committee for Clinical Research; Komisija Republike Slovenije za medicinsko etiko; Comitato Etico Indipendente Presso La Fondazione Ptv Policlinico Tor Vergata Di Roma; Comitato Etico Regione Calabria Sezione Centro c/o A.O.U. Mater Domini Di Catanzaro; Comitato Etico Azienda Ospedaliera Universitaria Di Cagliari; Comitato Etico Cardarelli-Santobono c/o Ao Cardarelli; Comitato Etico Per La Sperimentazione Clinica Delle Province Di Verona E Rovigo, Presso Aoui Verona; Eticka Komise Fn Brno; Eticka Komisia Dfnsp Bratislava; Eticka Komisia Pri Dfn Kosice; Eticka Komisia Bratislavskeho Samospravneho Kraja; Comisia Natională de Bioetică a Medicamentului şi a Dispozitivelor Medicale; Comitato Etico Milano area 1 c/o ASST FBF Sacco—P.O. L. Sacco; Comité de Ética de la Investigación de Centro Hospital Universitario Virgen del Rocío; Comité Ético de Investigación Clínica Fundació Sant Joan de Déu Generalitat de Catalunya. Departament de Salut; Comité Ético de Investigación Clínica Hospital Infantil Universitario Niño Jesús; Consejería de Sanidad Dirección General de Salus Pública Junta de Castilla León; Dirección General de Asistencia Sanitaria, Consejería de Sanidad Gobierno del Principado de Asturias; Dirección General de Planificación, Ordenación Sanitaria y Farmacéutica e Investigación, Consejeria de Sanidad y Política Social Región de Murcia; Ethics Committee at Moscow Institute of Pediatrics and Pediatric Surgery; Paula Stradiņa klīniskās universitātes slimnīcas, Attīstības biedrības Klīniskās izpētes Ētikas komiteja, Ethics Committee for Clinical Research; The First Affiliated Hospital of The Fourth Military Medical University; Zhongshan Hospital Fudan University.

## Author Contributions

AJ, EB, MB, PC, MD, JF, MF, CH, SJ, JL, AM, RN, VS, MS, RT, BZ, and JK: designing the study, patient accrual, clinical care, data interpretation, drafting, revising, final review, and approval of the manuscript. TC, VC, GBdA, PdV, CF, FO, JQ, YT, and SY: designing the study, data interpretation, drafting, revising, final review, and approval of the manuscript. LD: designing the study, trial management, data collection, data analysis, data interpretation, drafting, revising, final review, and approval of the manuscript. RM: designing the study, data analysis, data interpretation, drafting, revising, final review, and approval of the manuscript. SS: designing the study, trial statistician, data analysis, data interpretation, drafting, revising, final review, and approval of the manuscript.

### Conflict of Interest Statement

AJ, EB, TC, VC, PC, GBdA, PdV, JK, JF, MF, CF, CH, SJ, RN, FO, JQ, MS, RT, MD, JL, AM, SY, MB, and BZ received honoraria and support for the travels from Novartis. VC received personal fees for consulting, lecture fees and travel from Actelion, Bayer, Biogen Idec, Boehringer Ingelheim, Gilead, GSK, MSD, Novartis, Pfizer, Roche, Sanofi; grants from Actelion, Boehringer Ingelheim, GSK, Pfizer, Roche; personal fees for developing educational material from Boehringer Ingelheim and Roche. PdV has been on the study steering group of the EXIST-1, 2, and 3 studies sponsored by Novartis, and co-PI on two investigator-initiated studies part-funded by Novartis. RN received grant support, paid to her institution, from Eisai and lectures fees from Nutricia, Eisai, Advicenne, and GW Pharma. YT received personal fee from Novartis for lecture and for copyright of referential figures from the journals, and received grant from Japanese government for intractable epilepsy research. SJ was partly financed by the EC Seventh Framework Programme (FP7/2007–2013; EPISTOP, grant agreement no. 602391), the Polish Ministerial funds for science (years 2013-2018) for the implementation of international cofinanced project and the grant EPIMARKER of the Polish National Center for Research and Development No STRATEGMED3/306306/4/2016. JK, PC, CH, JL, and JQ received research grant from Novartis. RM and SS are employees of Novartis. LD was Novartis employee at the time of manuscript concept approval. This study was funded by Novartis Pharma AG. All authors approved the final version of the manuscript prior to submission.

## TOSCA Investigators

**Japan:** Nobuo Shinohara, Shigeo Horie, Masaya Kubota, Jun Tohyama, Katsumi Imai, Mari Kaneda, Hideo Kaneko, Yasushi Uchida, Tomoko Kirino, Shoichi Endo, Yoshikazu Inoue, Katsuhisa Uruno; **Turkey:** Ayse Serdaroglu, Zuhal Yapici, Banu Anlar, Sakir Altunbasak; **Russia:** Olga Lvova, Oleg Valeryevich Belyaev, Oleg Agranovich, Elena Vladislavovna Levitina, Yulia Vladimirovna Maksimova, Antonina Karas; **China:** Yuwu Jiang, Liping Zou, Kaifeng Xu, Yushi Zhang, Guoming Luan, Yuqin Zhang, Yi Wang, Meiling Jin, Dingwei Ye, Weiping Liao, Liemin Zhou, Jie Liu, Jianxiang Liao, Bo YAN, Yanchun Deng, Li Jiang, Zhisheng Liu, Shaoping Huang, Hua Li; **Korea:** Kijoong Kim; **Taiwan:** Pei-Lung Chen, Hsiu-Fen Lee, Jeng-Dau Tsai, Ching-Shiang Chi, Chao-Ching Huang; **Australia:** Kate Riney, Deborah Yates, Patrick Kwan; **Thailand:** Surachai Likasitwattanakul, Charcrin Nabangchang, Lunliya Thampratankul Krisnachai Chomtho, Kamornwan Katanyuwong, Somjit Sriudomkajorn; **South Africa:** Jo Wilmshurst; **Israel**: Reeval Segel, Tal Gilboa, Michal Tzadok, Aviva Fattal- Valevski; **Greece:** Panagiotis Papathanasopoulos, Antigone Syrigou Papavasiliou, Stylianos Giannakodimos, Stylianos Gatzonis, Evangelos Pavlou, Meropi Tzoufi; **Netherlands:** A.M.H. Vergeer; **Belgium:** Marc Dhooghe, Hélène Verhelst, Filip Roelens, Marie Cecile Nassogne, Pierre Defresne, Liesbeth De Waele, Patricia Leroy, Nathalie Demonceau, Benjamin Legros, Patrick Van Bogaert, Berten Ceulemans, Lina Dom; **France:** Pierre Castelnau, Anne De Saint Martin, Audrey Riquet, Mathieu Milh, Claude Cances, Jean-Michel Pedespan, Dorothee Ville, Agathe Roubertie, Stéphane Auvin, Patrick Berquin, Christian Richelme, Catherine Allaire, Sophie Gueden, Sylvie Nguyen The Tich, Bertrand Godet; **Spain**: Maria Luz Ruiz Falco Rojas, Jaume Campistol Planas, Antonio Martinez Bermejo, Patricia Smeyers Dura, Susana Roldan Aparicio, Maria Jesus Martinez Gonzalez, Javier Lopez Pison, Manuel Oscar Blanco Barca, Eduardo Lopez Laso, Olga Alonso Luengo, Francisco Javier Aguirre Rodriguez, Ignacio Malaga Dieguez, Ana Camacho Salas, Itxaso Marti Carrera, Eduardo Martinez Salcedo, Maria Eugenia Yoldi Petri, Ramon Cancho Candela; **Portugal**: Ines da Conceicao Carrilho, Jose Pedro Vieira, José Paulo da Silva Oliveira Monteiro, Miguel Jorge Santos de Oliveira Ferreira Leao, Catarina Sofia Marceano Ribeiro Luis, Carla Pires Mendonca; **Lithuania:** Milda Endziniene; **Latvia:** Jurgis Strautmanis; **Estonia:** Inga Talvik; **Italy:** Maria Paola Canevini, Antonio Gambardella, Dario Pruna, Salvatore Buono, Elena Fontana, Bernardo Dalla Bernardina; **Romania:** Carmen Burloiu, Iuliu Stefan Bacos Cosma, Mihaela Adela Vintan, Laura Popescu; **Czech Republic:** Karel Zitterbart; **Slovakia:** Jaroslava Payerova, Ladislav Bratsky, Zuzana Zilinska; **Austria:** Ursula Gruber-Sedlmayr, Matthias Baumann, Edda Haberlandt, Kevin Rostasy, Ekaterina Pataraia; **United Kingdom:** Frances Elmslie, Clare Ann Johnston, Pamela Crawford; **Denmark:** Peter Uldall; **Sweden:** Paul Uvebrant, Olof Rask; **Norway:** Marit Bjoernvold, Eylert Brodtkorb, Andreas Sloerdahl, Ragnar Solhoff, Martine Sofie Gilje Jaatun; **Poland:** Marek Mandera, Elzbieta Janina Radzikowska, Mariusz Wysocki; **Germany:** Michael Fischereder, Gerhard Kurlemann, Bernd Wilken, Adelheid Wiemer-Kruel, Klemens Budde, Klaus Marquard, Markus Knuf, Andreas Hahn, Hans Hartmann, Andreas Merkenschlager, Regina Trollmann.
